# Effectiveness and Safety of Pembrolizumab in Recurrent and Relapsed Classic Hodgkin Lymphoma: A Systematic Review

**DOI:** 10.7759/cureus.46032

**Published:** 2023-09-26

**Authors:** Md Fahad Hossain, Manish Kharel, Mahfuza Akter, Bibek Parajuli, Indresh Yadav, Nitesh Mandal, Anjali Mandal, Syed Nurul Aziz

**Affiliations:** 1 Ministry of Health, Kishoreganj Upazila Health Complex, Kishoreganj, BGD; 2 Medicine and Surgery, Jahurul Islam Medical College Hospital, Bhagalpur, BGD; 3 Medicine, Sylhet MAG (Muhammad Ataul Goni) Osmani Medical College, Sylhet, BGD; 4 Internal Medicine, Gandaki Medical College, Pokhara, NPL; 5 Internal Medicine, Samar Hospital and Research Center Pvt. Ltd., Janakpur, NPL; 6 Internal Medicine, Community Based Medical College, Mymensingh, BGD; 7 Internal Medicine, Jahurul Islam Medical College, Bhagalpur, BGD; 8 Medicine and Surgery, Chitwan Medical College, Bharatpur, NPL; 9 Obstetrics and Gynaecology, University of Missouri, Columbia, USA; 10 Internal Medicine, Shaheed Suhrawardy Medical College, Dhaka, BGD

**Keywords:** efficacy, safety, recurrent and relapsed chl, immune checkpoint inhibitor, pembrolizumab, classical hodgkin lymphoma (chl)

## Abstract

Classical Hodgkin lymphoma (cHL) has achieved high cure rates as a result of recent advancements in treatment. However, recurring or relapsed illness still poses a therapeutic challenge. Immune checkpoint inhibitor pembrolizumab, which targets PD-1, is now being commonly used as part of immunotherapy for recurrent and relapsed cHL. We found eight appropriate articles through systematic search and conducted in-depth analysis to find insights into the effectiveness and safety profiles of pembrolizumab by analyzing clinical trial data in patients with recurrent and relapsed cHL. Analysis of the studies shows that response rates, progression-free survival, and patient-reported quality of life have all significantly improved. However, immune-related consequences are among the adverse outcomes. The necessity for continued study is highlighted by the variation in reported adverse events and follow-up times. Clinicians, researchers, and other healthcare professionals can use this study as a resource to provide knowledgeable and individualized patient care in cHL.

## Introduction and background

A tumor of the lymphatic system known as classic Hodgkin lymphoma (cHL) is defined by the presence of abnormal Reed-Sternberg cell populations in the presence of inflammatory lymphocytes. Although there is currently a high cure rate for cHL, up to 30% of patients in the advanced stages and 5-10% of those in the limited stage experience relapses [1-4]. Immunotherapy has become a potential option for treating cHL in these complex situations in recent years.

Recent United States Food and Drug Administration (FDA) approval of the monoclonal antibody known as pembrolizumab, which targets the programmed cell death protein 1 (PD-1), has totally altered the landscape of the treatment of relapsed or resistant cHL [5]. By inhibiting the link between PD-1 and its two ligands (PD-L1 and PD-L2), pembrolizumab effectively restores the body's natural defense systems and, as a result, increases the body's defenses against malignant cells [6]. The importance of this mechanism of action in cHL is highlighted by the fact that the tumor microenvironment frequently uses the PD-1 pathway to avoid immune monitoring [6].

There are significant clinical problems associated with the treatment of recurrent or relapsing cHL, although beneficial in some circumstances, traditional salvage chemotherapy regimens are accompanied by significant toxicity and may not be appropriate for many patients [7]. Pembrolizumab's introduction has created a new path for individuals with refractory illness, promising better outcomes with a good safety profile [7].

Pembrolizumab's safety and effectiveness in treating recurrent and relapsed cHL have been studied in numerous clinical trials [8-15]. This research has produced optimistic findings, leading to regulatory approvals and altering the landscape of available treatments. Pembrolizumab, however, is not without potential hazards and restrictions, just like any treatment. There have been reports of immune-related adverse events (irAEs), demanding careful surveillance and management techniques [16]. Additionally, research is still being done on the best order to combine pembrolizumab with other therapy modalities, predictive biomarkers, and patient selection [16].

This in-depth analysis explores pembrolizumab's safety and effectiveness in treating recurrent and relapsed cHL. We seek to provide an in-depth understanding of the advantages and difficulties associated with this immunotherapeutic strategy by compiling and critically reviewing current research. The evaluation will cover important elements like the results of clinical trials, response rates, response times, overall survival rates, and adverse event profiles. As part of the inquiry, possible biomarkers that can help with patient selection and therapy personalization will also be found.

It is crucial to critically evaluate immunotherapy's relevance in particular disease contexts as it is incorporated more and more into the toolbox of oncology. Pembrolizumab's effectiveness in treating recurrent and relapsing cHL has inspired optimism, but therapeutic decision-making needs to be supported by a thorough awareness of its advantages, constraints, and potential hazards. This study seeks to provide a thorough understanding of pembrolizumab's significance in the emerging field of cHL therapy for clinicians, scientists, and healthcare professionals. This review seeks to contribute to informed and individualized patient care in the area of recurrent and relapsed cHL by synthesizing information, analyzing molecular insights, and addressing key clinical factors.

## Review

Methodology

Preferred Reporting Items for Systematic Reviews and Meta-analysis (PRISMA) guidelines were followed to do this systematic review [17].

Data Sources

On July 28, 2023, the following databases were searched: MEDLINE (Medical Literature Analysis and Retrieval System Online) through Pubmed, The Cochrane Library (Cochrane Central Register of Controlled Trials (CENTRAL)), EBSCOhost, Web of Science, and Scopus. The keywords used were Pembrolizumab, SCH-900475, pembrolizumab, MK-3475, Keytruda, Hodgkin Disease, Hodgkin Lymphoma, Hodgkin Granuloma, Malignant Lymphoma, etc.

Study Selection

We selected articles that examined pembrolizumab's advantages and disadvantages when used to treat cHL that had recurred or relapsed. We looked at comparative cross-sectional research, case-control studies, cohort studies, randomized controlled trials (RCTs), and non-randomized trials.

We excluded studies that didn't take pembrolizumab into account; studies that weren't about lymphoma, lab experiments, unfinished or ongoing investigations, animal studies, reviews, case series, case reports, letters, comments, editorials, book chapters, and opinions were all disqualified. 

Two reviewers each examined the title and abstract of the papers that were redeemed, and any inconsistencies were settled by a lead reviewer. To filter the articles, we used the internet tool Rayyan (Rayyan Systems, Inc., Cambridge, Massachusetts, United States) [18]. Then, two distinct teams looked over the full-text papers, with the lead reviewer resolving any conflicts. Utilizing the "prioritization and sequential exclusion" method, we eliminated articles that did not fit in our criteria [19]. Exclusion grounds were disclosed.

Data Extraction

We gathered information about the study population, the duration and dosage of the intervention, the positive and negative results, the gravity of the bad results, and limitations. Two reviewers separately extracted data, and the main reviewer cross-checked it to settle any disagreements.

Data Analysis

A narrative synthesis was completed. The lack of sufficient data precluded the use of a meta-analysis.

Assessment of Risk of Bias

Using the Risk of Bias in Non-Randomised Studies - of Interventions (ROBINS-I) tool in non-randomized trials and the risk of bias (ROB) 2.0 tool, the ROB in randomized trials was assessed [20-21]. The ROB was evaluated independently by two teams of review authors, and any disagreements were settled by discussion. 

Results

Search Results

**Figure 1:**
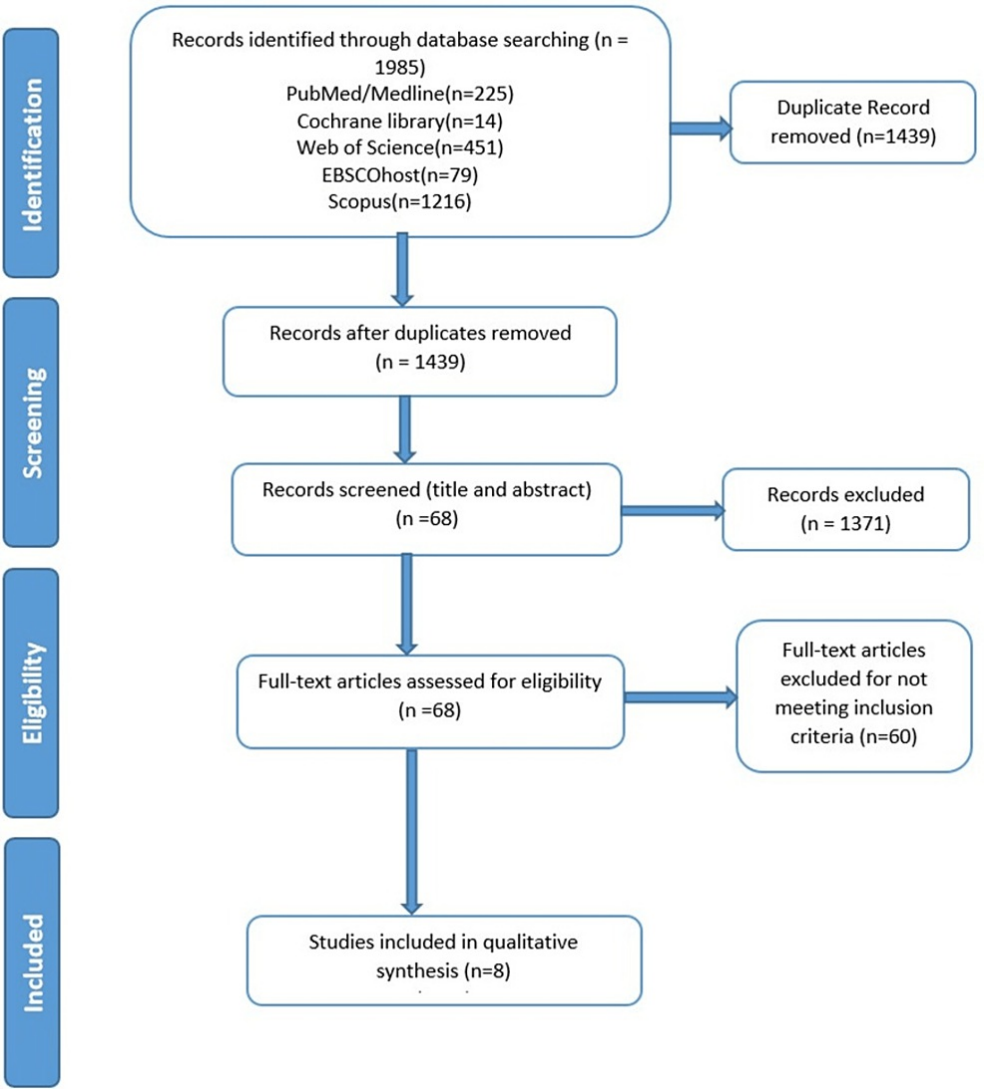
PRISMA flow diagram depicting the study selection process PRISMA: Preferred Reporting Items for Systematic Reviews and Meta-Analyses

A thorough search across five databases turned up 1985 articles. We listed 1439 articles for title and abstract screening after deleting 546 duplicates. A total of 1371 items were eliminated at this point for failing to meet the inclusion criteria listed in the methodology section. Sixty of the remaining articles were disqualified at the full-text screening stage because they weren't pertinent to the research intervention. The analysis finally comprised one RCT and seven clinical studies. The detailed PRISMA diagram of the article inclusion process is shown in Figure 1.

ROB Assessments of Clinical Trials

**Figure 2:**
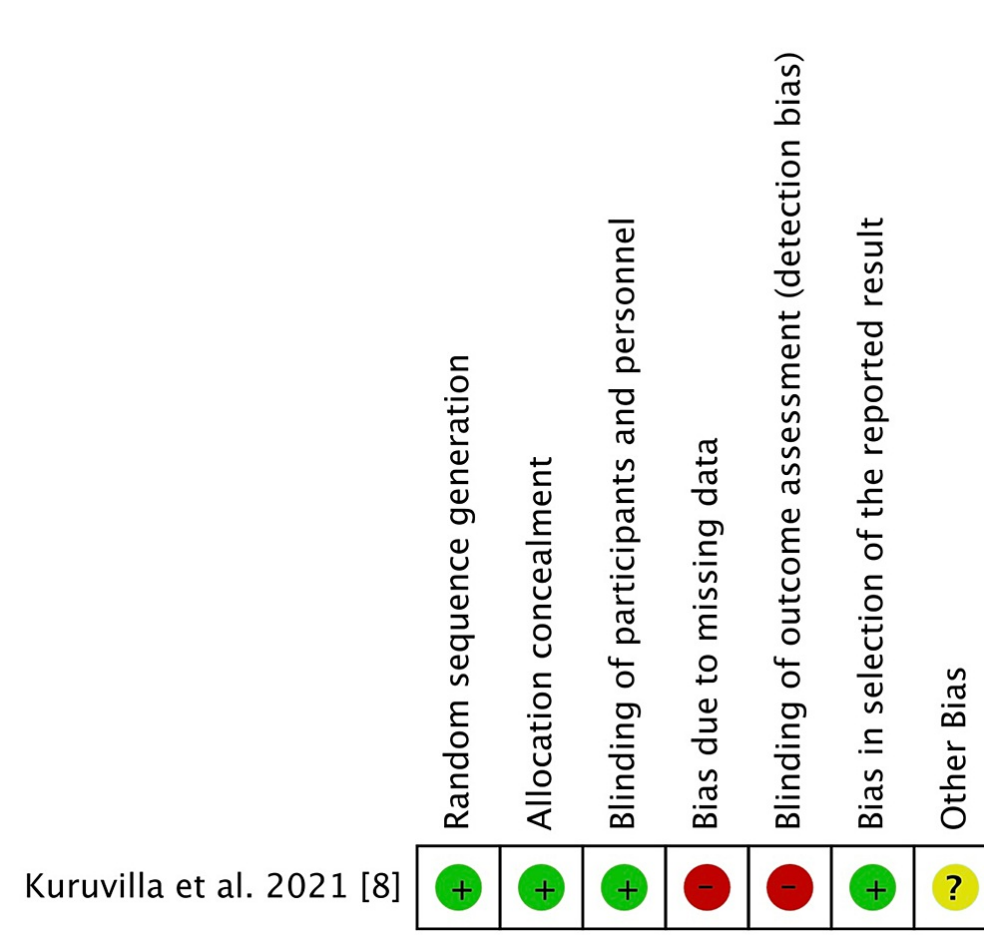
Risk of bias assessment outcome of a randomized trial

**Figure 3:**
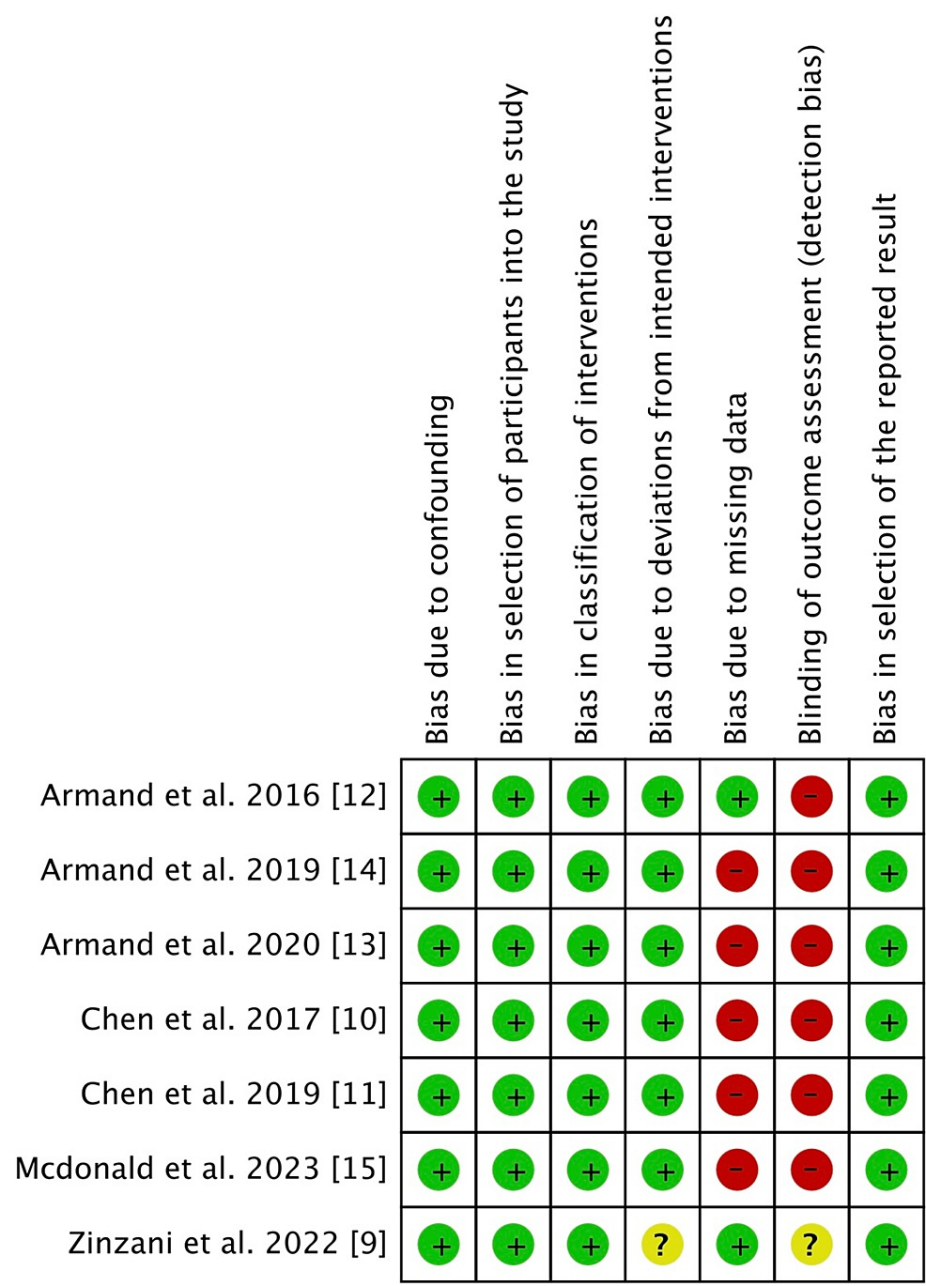
Risk of bias assessment of non-randomized trials

An open-level randomized experiment was conducted by Kuruvilla et al. [8]. Bias resulting from lack of blinding is therefore significant. Regarding any further biases, nothing is disclosed. Four out of every six non-randomized studies contain missing outcome data of higher than 10%. So there is a considerable chance of bias. The likelihood of bias is considerable for this component since five studies did not apply blinding for outcome measurement. Figure 2 and Figure 3 show the graphical representation of randomized and non-randomized trials, respectively.

Characteristics of Included Studies

Except for one Phase 3 study led by Kuruvilla et al. [8], most of the studies used a single-arm cohort design. High cure rates for cHL have been achieved, thanks to recent advancements in treatment, but recurrent or relapsing illness is still a clinical problem [8]. A novel treatment for these conditions has emerged: pembrolizumab, an immune checkpoint inhibitor that targets PD-1 [22]. Pembrolizumab stimulates the immune system and prevents PD-1 from interacting with its ligands to fight against cancer cells. This is especially important for people with cHL because cancers frequently use immune evasion techniques. Studies show that response rates, progression-free survival, and patient-reported quality of life have all significantly improved. However, immune-related consequences are still among the adverse outcomes. The necessity for continued study is highlighted by the variation in reported adverse events and follow-up times. Pembrolizumab shows obvious promise in the management of cHL, highlighting the significance of thorough comprehension, monitoring, and specialized care. This review, which uses a randomized controlled approach with brentuximab vedotin as the control, aims to evaluate the efficacy and safety of pembrolizumab in case of refractory and relapsed cHL. It is a resource for clinicians, researchers, and healthcare professionals and contributes to informed and individualized patient care in cHL. These trials, which took place between 2016 and 2023, had 636 individuals with a median age of 32 [8-15]. The studies span multiple countries and both sexes. It should be noted that Chen et al. and Armand et al. were follow-up extension studies that were incorporated to demonstrate the long-term outcomes of the earlier trials [11,13]. In addition, Zinzani et al. and Chen et al. used the European Organisation for Research and Treatment of Cancer (EORTC) Quality of Life Questionnaire Core 30 to evaluate the patient-reported outcome along with clinical results, enabling a thorough assessment of the treatment's effect on quality of life [9,11].

Description of Intervention

Except in the Mcdonald et al.'s [15] study and Armand et al.'s [12] phase 1b studies, where pembrolizumab is provided at 400 mg every six hours and 10 mg/kg twice weekly, respectively, the majority of studies administered pembrolizumab 200 mg intravenously three times a week. Additionally, in the study by Kuruvilla et al., Brentuximab vedotin was administered intravenously at a rate of 1/8 mg/kg three times a week (with a maximum dose of 180 mg) [8]. Typically, pembrolizumab was given up to progression, intolerable toxicity, or discontinuation.

Description of Outcomes

Table 1 demonstrates the detailed outcome of the intervention with doses and a summary

**Table 1:** Characteristics of the included studies HSCT: Hematopoietic stem cell transplantationl PFS: Progression-free survival; BV: brentuximab vedotin; BICR: Blinded Independent Central Review; ORR: Objective response rate; PD: Progressive Disease; ASCT: Autologous stem-cell transplantation; OS: Overall survival; CRR: Complete response rate; DOR: Duration of response; HRQoL: Health-related quality of life; EORTC QoL Questionnaire Core 30 (QLQ-C30): The European Organisation for Research and Treatment of Cancer Quality of Life Questionnaire Core 30; EQ-5D: European Quality of Life Five Dimension; RR cHL: Refractory and Relapsed Hodgkin Lymphoma.

Study	Components	Features
Kuruvilla et al.,2021 [8] NCT02684292	Study design	Randomized phase 3 study, Open-label Multicentre
	Summary	In order to compare pembrolizumab with brentuximab in r/r cHL, 151 patients were administered pembrolizumab, and 153 patients were administered Brentuximab vedotin. The phrase "interim analysis" is used here which took 25.7 months on average from randomization to data cutoff. PFS analysis and OS were the two primary objectives. Secondary objectives were objective response, full remission, and durability of response as well as progression-free survival after autologous or allogeneic HSCT, excluding clinical and imaging data. When compared to brentuximab vedotin in this study, Pembrolizumab is statistically and clinically effective in r/r cHL evidenced by the increase in PFS
	Population	304 patients who were ineligible for or had experienced a relapse following autologous hematopoietic stem cell transplantation were assigned at random (151 in pembrolizumab, 153 in brentuximab vedotin). Patients with non-responsive illness who might become sensitive following treatment, enabling them to proceed to transplantation; patients who received autologous HSCT but relapsed; and patients who had relapsed after autologous HSCT are the subpopulations examined in this study.
	Intervention	200 mg of pembrolizumab was injected intravenously every three weeks. Every three weeks, 180 mg of brentuximab vedotin was delivered intravenously (1/8 mg/kg).
	Outcome	The PFS in pembrolizumab was 13.2 months compared to brentuximab vedotin which was 8.3 months (P: 0.0027). In addition, Secondary PFS was 12 months in the pembrolizumab group, while brentuximab vedotin was 8 months. The median duration of response in pembrolizumab vs. brentuximab vedotin was 20 months vs. 13.8 months. Moreover, the pembrolizumab group vs. brentuximab vedotin group for CR was 26% vs. 24%, PR was 42% vs. 37%, objective response was 65.6% vs. 83% (BICR), 68.2% vs. 60.1% (investigator review). Pembrolizumab is superior to bv in cHL patients who recur after autologous HSCT (PFS: 14.7 vs. 10.8) and also to those who don't fulfill criteria for autologous HSCT (PFS: 12.5 vs. 5.7) following subgroup analysis
	Limitation	Three subpopulations with various treatment modalities for which the next most effective course of action has not yet been determined Treatment variation
Zinzani et al.,2022 [9] NCT02684292	Study design	Randomized phase 3 study, Open-label Multicentre
	Summary	In order to quantify health-related quality of life (HRQoL) the PROs from the KEYNOTE-204 using the EORTC QoL Questionnaire Core 30 (QLQ-C30) and the EuroQoL EQ-5D, which were taken at baseline, once every six weeks until 24th week, and after once every 12 weeks. Pembrolizumab showed overall improvements in HRQoL measures compared to BV.
	Population	Random assignments of 304 patients among which 153 in brentuximab vedotin and 151 in pembrolizumab
	Intervention	200 mg of pembrolizumab was injected intravenously every three weeks. Every three weeks, 1/8 mg/kg of the anti-Brentuximab vedotin drug was intravenously delivered; the highest dose was 180 mg.
	Outcome	The study showed significant improvement in global health status (GHS)/quality of life (QoL) (GHS/QoL: 8.60 [ P =.0004] in all domains except mental functioning and emotion. In addition, TTD was significantly prolonged in Pembrolizumab compared to BV ( HR:0.4, P:0.003).
	Limitation	Open-label trial design Absence of hypothesis for HRQoL endpoints
Chen et al., 2017 [10] NCT02453594,	Study design	Phase II single-arm study Multi-cohort, Multicentre
	Summary	210 individuals with r/r cHL were studied to determine the safety and effectiveness of pembrolizumab. The ORR by BICR was the primary endpoint, and the investigator review and OS, PFS, CRR, DOR by BICR, and investigator assessment were the secondary endpoints. According to this study, pembrolizumab has a high rate of response and lowers tumor burden
	Population	210 individuals, including 60 in Cohort 3, 69 in Cohort 1, and 80 in Cohort 2 which is based on lymphoma progression (1)BV following ASCT ; (2) BV following salvage chemotherapy; and (3) only ASCT
	Intervention	200 mg of Pembrolizumab intravenously once every three weeks.
	Outcome	The ORR was 69% and the overall CRR was 22.4%. The maximum reduction in tumor burden was detected mainly in the initial evaluation. Additionally, the PFS rate at 6 months was 72.4% and the OS rate was 99.5%. Within six months, 75.6% of the respondents responded. The global health status/quality of life score and EQ-5D visual analog score have increased from baseline to week 12 across all cohorts. On subgroup analysis: Patients who had undergone at least three lines of therapy and less than three lines of therapy both had similar ORRs (71.4% vs. 68.7%).ORR was 79.5% (95% CI, 68.4% to 88.0%).In the analysis of the 73 primary refractory patients, ORR was 79.5%. Similarly, In patients (n = 35) who had never had BV before, the ORR was 71.4%.
	Limitation	Short follow-up period No control group
Chen et al.,2019 [11] Follow-up study of NCT02453594.	Study design	Phase II single-arm study Multi-cohort, Multicentre
	Summary	The phase 2 study has an extra follow-up of approximately 17.5 months in which a longer response of pembrolizumab in r/r cHL was assessed. The ORR by BICR was the primary endpoint, and the investigator review and OS, PFS, CRR, DOR by BICR, and investigator assessment were the secondary endpoints. Pembrolizumab is effective against r/r cHL with a good safety profile.
	Population	210 individuals, including 60 in Cohort 3, 69 in Cohort 1, and 80 in Cohort 2 which is based on lymphoma progression (1)BV following ASCT ; (2) BV following salvage chemotherapy; and (3) only ASCT
	Intervention	200 mg of Pembrolizumab intravenously once every three weeks.
	Outcome	According to our study, the ORR by BICR for all patients was 71.9%, CRR was 27.6%, and PR rate of 44.3%. The TTR was 2.8 months which was the same for patients in each group. In addition, the DOR was 16.5 months. 58.5% of the responses lasted under 12 months, while 42.5% of the responses lasted under 24 months. All patients had a PFS of 13.7 months. Additionally, the CRR and ORR in individuals who received a second treatment of pembrolizumab are 50% and 75%, respectively
	Limitation	No control group. Short follow-up period
Armand et al.,2016 [12] NCT01953692	Study design	Phase Ib open-label trial Multicohort
	Summary	31 individuals with r/r cHL disease were taken in the study. The patient's response to the treatment was evaluated after 12 weeks and after that every 8 weeks. Safety and the percentage of complete remission (CR) served as the main objectives. In this trial, pembrolizumab-PD-1 blocking was linked to a high overall response rate, a good safety profile, and some signs of tumor reduction after treatment.
	Population	31 patients have classic HL that has relapsed or is resistant. Patients received pembrolizumab treatment, and brentuximab vedotin treatment, and did not fulfill criteria for or did not consent for ASCT or had relapsed or refractory illness.
	Intervention	Pembrolizumab is administered intravenously at 10 mg/kg once every two weeks
	Outcome	The CRR was 16%, ORR was 65% and partial remission rate was 48%. 70 % of responses lasted more than 24 weeks, the progression-free survival rate at 24 weeks and 52 weeks, and the overall survival rate was 69%,46%, and 100 % respectively
	Limitation	The sample size was relatively small. Long-term follow-up could only be carried out after a period of time.
Armand et al.,2020 [13] NCT01953692	Study design	Phase Ib open-label trial Multicohort
	Summary	Results after four years are provided in order to comprehend the resilience of reactions. Safety and CR rate were the main outcomes.PFS, DOR, ORR, and OS were considered secondary goals. (CR, PR), responses (stable disease, PD), and partial responses (CR, PR).
	Population	31 patients have classic HL that has relapsed or is resistant. Patients received pembrolizumab treatment and brentuximab vedotin treatment, and either did not fulfill criteria or did not give consent for autologous stem cell transplantation (ASCT), or had relapsed or refractory illness.
	Intervention	Pembrolizumab at 10 mg/kg is administered intravenously once every two weeks.
	Outcome	58% ORR, 19% CR, and 39% PR were attained. The Kaplan-Meier approach showed 50% DOR rates at 24 and 36 months. Also not met was the median overall survival; the 36-month overall survival rate was 81%.
	Limitation	Small sample size
Armand et al.,2019 [14]	study design	Phase 2 open-label, multicenter, multicohort, investigator-initiated research study
	Summary	Pembrolizumab was given to 31 patients beginning within 21 days of their release from the hospital following their ASCT to see the outcome of the procedure after giving pembrolizumab. Patients with RR cHL were successfully treated with pembrolizumab as post-ASCT consolidation, which produced a significant PFS.
	Population	Thirty-one patients with r/r cHL
	Intervention	Every three weeks, a fixed dose of 200 mg of pembrolizumab is given intravenously.
	Outcome	The 19-month time PFS was 81% overall and OS was 100%. The 19-month PFS who had more than one of the five risk factors was 85% and 83% among patients with more than two high-risk variables).
	limitation	Small sample size. Open-label study with no control.
Mcdonald et al.,2023 [15]	Study design	Nonrandomized trial.
	Summary	The Study demonstrates the effectiveness and safety of pembrolizumab, 60 R/R CHL were included. The primary goal was to obtain ORR . The secondary goals were safety and DOR.Other Findings were OS and PFS. Pembrolizumab exhibits significant anticancer activity in R/R cHL patients follow-up after nine months..
	Population	60 patients have relapsed and refractory cHL
	Intervention	400 mg pembrolizumab administered once in every 6 weeks.
	Outcome	In our study ORR was 65%, 31.7% was PR, and 33.3% was CR
	limitation	Small sample size Short follow-up period

Effectiveness

In a comparison study between pembrolizumab and brentuximab vedotin, Kuruvilla et al. found that pembrolizumab significantly increased progression-free survival and median duration of response [8]. Additionally, Zinzani et al. [9] assessed patient-reported outcomes from Kuruvilla et al.'s study [8] and found that, while brentuximab vedotin showed worsening scores at 24 weeks, pembrolizumab improved quality of life. Pembrolizumab showed significant response rates with tumor burden reduction, a better quality of life score, and a high response rate in patients who were difficult to treat, according to Chen et al.'s multi-cohort phase 2 trial [10]. High and significant overall and complete response rates were found in the study, and this promising trend continued over a two-year follow-up period [11]. In a study on patients with relapsed or resistant cHL, McDonald et al. demonstrated potent anticancer efficacy. Overall and complete response rates were statistically significant, according to the study [15]. Armand et al. also showed effective anticancer activity with a significant complete response rate and overall response rate [12]. A four-year follow-up provided more evidence for this, maintaining the same findings [13], and additionally, demonstrated promising progression-free survival (PFS).

Safety

When compared to brentuximab vedotin, pembrolizumab had a greater incidence of moderate to severe irAEs pneumonitis, according to Kuruvilla et al. [8]. The pembrolizumab group also frequently experiences peripheral neuropathy, hypothyroidism, reduced neutrophil count, and neutropenia. In Chen et al.'s study, irAEs affected 28.6% of patients, while AEs related to treatment included pyrexia (10.5%) and hypothyroidism (12.4%). Pembrolizumab's safety profile was constant over a two-year follow-up period, with low-grade hypothyroidism serving as the predominant irAE (15.7%) [11]. Forty percent of R/R cHL patients had adverse events related to drugs, and 5% of those had moderate AEs, according to McDonald et al. [15]. Armand et al. describe pneumonitis (10%), nausea (13%), hypothyroidism (16%), and diarrhea (16%) [12]. Sixteen percent of individuals had severe (grade 3) AEs. Treatment-related AEs persisted throughout long-term follow-ups, with diarrhea hypothyroidism affecting 23% and 13% of patients, respectively. Additionally, different grades of transaminitis (10%), diarrhea (3%), hypothyroidism (7%), neutropenia (7%), leukopenia (13%), and immune-related symptoms such as pneumonitis (most frequent) were identified in the Armand et al.'s phase 2 research [14].

Discussion

Summary of Findings

A thorough grasp of the efficacy and consequences of pembrolizumab treatment in relapse and recurrent classic Hodgkin lymphoma (HL) across diverse situations is provided by the findings of the systematic review which included a number of studies. Phase 1b research was undertaken by Armand et al. to examine pembrolizumab's effectiveness in patients who were substantially pretreated and had failed brentuximab vedotin therapy [12]. The trial demonstrated the significant complete response rate and overall response rate demonstrating long-term response with single-agent pembrolizumab. A four-year follow-up supported this, with a constant significant complete response rate and overall response rate demonstrating persistent antitumor activity [13]. In a Phase 2b study conducted by Armand et al., pembrolizumab was used as a consolidation drug after transplantation. This experiment showed that using pembrolizumab as consolidation therapy enhanced the response to transplantation [14].

Chen et al.'s phase 2 study displayed high and significant response rates with pembrolizumab accompanied by tumor burden reduction (more than 90%) and an improved quality of life score across all cohorts [10,11]. Moreover, a high response rate was also achieved in primary refractory illness (35%) and also in patients not taking brentuximab vedotin (71.4%). This positive trend persisted over the follow-up study, with a consistently high and significant overall response rate and a complete response rate in refractory and relapsed Hodgkin lymphoma patients [11]. It also revealed that pembrolizumab has potent anticancer activity in R/R cHL patients who were brentuximab vedotin-naive (77.1%), as well as in those who received brentuximab vedotin before (70.6%) or after autologous stem-cell transplantation (ASCT) (80%). Kuruvilla et al. conducted a direct comparison of pembrolizumab and brentuximab vedotin, finding that pembrolizumab is more effective than brentuximab vedotin due to significantly improved progression-free survival (13.2 vs. 8.3 months), duration of response and objective response rate [8]. The longer-term advantage of pembrolizumab is demonstrated by an increase in response duration [23]. According to this review, Pembrolizumab should be the first choice of treatment for patients who don't fulfill the criteria for autologous hematopoietic stem cell transplantation (HSCT) or who have recurrence fter autologous HSCT.

Moreover, Zinzani et al. evaluated patient-reported outcomes from the study by Kuruvilla et al. indicating that quality of life improved with pembrolizumab, as demonstrated by an improved quality of life score at 24 weeks, while brentuximab vedotin showed worsening scores [8,9].

McDonald et al. conducted a non-randomized trial that was consistent with earlier research and demonstrated strong anticancer activity (objective response rate (ORR): 65%) [15].

The combined studies under consideration provide information on the safety profiles and side effects of pembrolizumab [24]. The study revealed tolerable side effects such as pneumonitis, hypothyroidism, and diarrhea are common in patients taking pembrolizumab. In addition, neutropenia was a common hematological finding in individuals on pembrolizumab. Notably, no cases of serious adverse events with potentially fatal outcomes were reported during the course of these investigations, indicating that pembrolizumab is a secure medication.

Pembrolizumab has notable response rates, a significant reduction in tumor burden, and improves the quality of life while maintaining a favorable safety profile, according to the in-depth research carried out in this study. These findings improve our comprehension of therapeutic strategies and emphasize the need for additional research to confirm their viability and deepen our knowledge of the potential advantages and disadvantages of pembrolizumab.

Agreement and Disagreement with Contemporary Research

There is both consensus and disagreement in the most recent study on pembrolizumab's efficacy and safety in the treatment of relapsed or resistant cHL [25]. Pembrolizumab is consistently described as being extremely efficacious in treating cHL in many researches, including those by Armand et al., Chen et al., and McDonald et al [10,12,15]. Impressive overall response rates and significant complete response rates are reported, suggesting durable antitumor activity in patients who have received a lot of prior treatment [10-15]. Pembrolizumab also increases progression-free survival when compared to alternative therapies, which further supports its potential to be a preferred option in this situation, according to Kuruvilla et al. and Armand et al. [8,12]. Pembrolizumab can improve the quality of life related to one's health, according to Zinzani et al.'s analysis of patient-reported outcomes [9].

There is, however, one significant area where there is disagreement: the safety profile [25]. While the studies generally agree that the side effects of pembrolizumab are manageable, they disagree on particular adverse occurrences. While other researchers did not highlight this as a major problem, Kuruvilla et al. observed a greater prevalence of grade 3-5 pneumonitis with pembrolizumab, suggesting differences in the reporting and interpretation of adverse events [8]. Additionally, the studies show variation in the length of follow-up. Questions have been raised concerning the ideal time frame for thoroughly evaluating the efficacy and safety of pembrolizumab because Chen et al. indicate positive trends over a two-year follow-up while Armand et al. retain consistent results over a lengthier four-year follow-up [11,14]. In conclusion, current research shows the promise of pembrolizumab in the treatment of cHL but also points out differences in the reporting of adverse events and the length of follow-up, requiring more study and customized treatment choices [26-29].

Research Implications

The conclusions from the thorough review have important ramifications for clinical practice and future directions for research [25,26]. These implications can help decision-makers and direct more research in a number of important areas.

First off, the study highlights pembrolizumab's potential as a top therapeutic choice for individuals with relapsed or resistant cHL. High response rates, better progression-free survival, and the ability to improve patient quality of life are all shown by it [8-15]. When choosing a course of treatment, doctors should take these outcomes into account, especially for patients who have experienced a relapse after receiving an autologous stem cell transplant (ASCT) or who are ineligible for ASCT. Future research should focus on identifying particular patient subgroups that can benefit the most from pembrolizumab therapy.

Second, although it has a controllable safety profile, pembrolizumab's side effects-including immune-related ones like pneumonitis and hypothyroidism-need to be proactively monitored and managed [8]. To ensure patient safety and treatment tolerability, healthcare professionals should continue to be cautious in identifying and managing these adverse effects. Strategies for improving the management of immune-related adverse events linked to pembrolizumab should be explored in further studies.

Thirdly, the disparity in follow-up times between studies emphasizes the need for more data collection to determine how long pembrolizumab's anticancer efficacy and safety profile will last [11,14]. The persistence of treatment responses and the possible appearance of late-onset adverse effects can both be learned through long-term follow-up studies. Particularly in the context of ASCT consolidation therapy, researchers should carry out thorough long-term follow-up studies.

As shown by enhanced EORTC Questionnaire Core 30 scores, pembrolizumab has a beneficial effect on the standard of life leading, which emphasizes the value of including feedback from patients in clinical study and everyday practice [8,9]. These metrics provide a comprehensive view of patient well-being and therapy efficacy, advancing our understanding of treatment outcomes.

Strengths and Limitations

We conducted this systematic study while rigorously following the PRISMA guidelines. Only RCTs were considered in this study. The Cochrane ROB evaluation method was used to thoroughly examine the bias risk of the listed articles. There are unquestionably some limitations in this review. Only English-language articles were considered. Studies that have been published in any other language may therefore have gone unnoticed. Second, we only incorporated a few RCTs with sufficient sample sizes. As a result, the results occasionally may be overstated.

## Conclusions

In this review, we looked into the shifting circumstances of the therapy of cHL, with an emphasis on the function of pembrolizumab in recurrent and relapsed cases. Pembrolizumab has completely changed how cHL is treated when it is refractory or relapses. Clinical trials have demonstrated its immune-boosting mechanism to be quite successful, leading to regulatory approvals and changes in treatment paradigms. High response rates, long-lasting effects, and enhanced progression-free survival, particularly in patients with few other therapy options, are important data that demonstrate pembrolizumab's efficacy. This places it in a position to be an effective therapy for people dealing with the difficulties of cHL. Despite irAEs, pembrolizumab has a tolerable safety profile, which emphasizes the significance of careful monitoring and effective management of AEs for patient safety. While variations and complexities exist in contemporary research, ongoing studies will continue to refine our understanding of pembrolizumab.
